# Adenomyoepithelioma with a human epidermal growth factor receptor 2-fluorescence in situ hybridization-confirmed ductal carcinoma in situ component: A case report and review of the literature

**DOI:** 10.1097/MD.0000000000022665

**Published:** 2020-10-16

**Authors:** Yusuke Amano, Mio Sakaguchi-Tamba, Yumiko Sasaki, Hisashi Oshiro, Noriyoshi Fukushima, Takashi Fujita, Shinobu Masuda, Toshiro Niki

**Affiliations:** a Department of Pathology; b Department of Breast Surgery, Jichi Medical University, Shimotsuke, Tochigi; c Division of Oncologic Pathology, Department of Pathology and Microbiology, Nihon University School of Medicine, Itabashi, Tokyo, Japan.

**Keywords:** adenomyoepithelioma, ductal carcinoma in situ, fluorescence in situ hybridization, human epidermal growth factor receptor 2, immunohistochemistry

## Abstract

**Introduction:**

Breast adenomyoepithelioma (AME) is a rare tumor composed of myoepithelial cells and ductal or luminal cells. Most cases of AME are benign, but rare cases in which either or both cell types exhibited malignant features have been reported. Due to its rarity, no diagnostic criteria for malignancy have been established for AME.

**Patient concerns:**

A 64-year-old woman presented with a mass in her right breast. Fine-needle aspiration cytology and biopsy examinations revealed lesions composed of spindle-shaped cells and round epithelial cells. AME was suspected, and partial mastectomy was performed.

**Diagnosis:**

The tumor specimen showed AME, which mainly consisted of spindle-shaped myoepithelial cells with slight atypia, admixed with tubular luminal cells and small areas of atypical intraductal proliferative lesions. No apparent features of malignancy, such as necrosis or invasion, were seen in the myoepithelial cells or the luminal or intraductal component. However, the atypical intraductal component exhibited focal nuclear atypia, a cribriform pattern, and moderate to strong membranous human epidermal growth factor receptor 2 (HER2) immunoreactivity. HER2 amplification was detected in focal regions of the atypical intraductal component by fluorescence in situ hybridization (FISH), which resulted in a diagnosis of AME with ductal carcinoma in situ.

**Outcomes:**

The patient did not receive further therapy and was free from tumor recurrence at 23 months after the operation.

**Conclusion:**

HER2 FISH might be useful for evaluating suspected AME tumors for malignancy when an atypical ductal lesion that lacks definitive features of malignancy is encountered.

## 1 Introduction

Adenomyoepithelioma (AME) of the breast was first reported by Hamperl in 1970.^[1]^ AME is composed of myoepithelial cells and a ductal or luminal component.^[1]^ Most cases of AME are benign, but rare malignant cases that displayed atypical histological features in either or both cell types have been reported.^[2–4]^ Malignant AME is defined as AME with carcinoma, including carcinoma derived from the luminal epithelium, carcinoma derived from the myoepithelium, and epithelial-myoepithelial carcinoma.^[2–5]^

Currently, there are no definitive histological criteria for diagnosing malignancy in atypical AME because of the rarity of the disease. The malignant tumor cells described in the literature were usually characterized by an invasive growth pattern, marked cytological atypia, the proliferation of atypical spindle-shaped myoepithelial cells, and an increased number of mitotic figures (>5/10 per high-power field).^[2,3,6]^ The cytological features described in malignant cases included nuclear enlargement; prominent nucleoli; and open, clumped chromatin in either the ductal epithelial or myoepithelial cells or both.^[2,3]^

Herein, we report a case of AME involving an atypical ductal proliferative lesion. In this case, human epidermal growth factor receptor 2 fluorescence in situ hybridization (HER2 FISH) demonstrated amplification of the HER2 gene in the atypical ductal component, which resulted in a diagnosis of AME with ductal carcinoma in situ (DCIS). To the best of our knowledge, this is the first case of malignant AME in which HER2 FISH provided an essential clue to establishing the diagnosis. HER2 FISH might be a useful method for assessing malignancy when AME with atypical ductal lesions is encountered.

## 2 Case presentation

**Figure 1:**
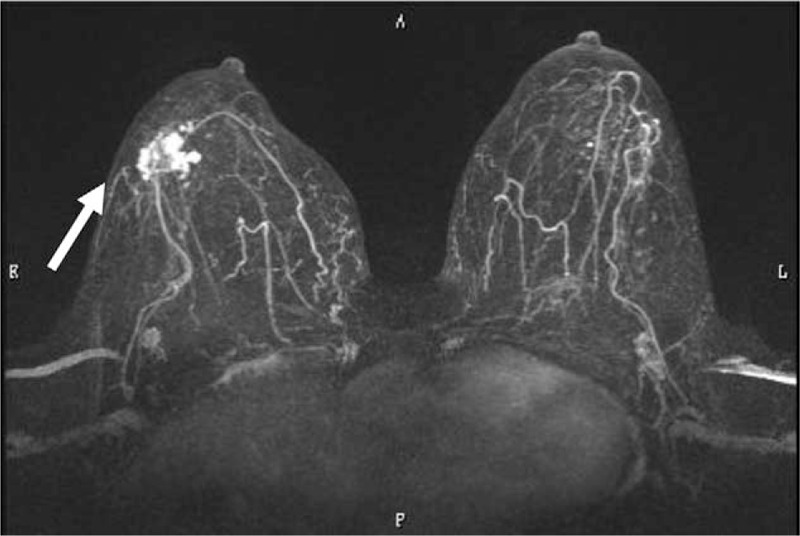
MRI findings. The horizontal view showed a nodular lesion (arrow), which measured 30 × 30 mm. MRI = magnetic resonance imaging.

**Figure 2:**
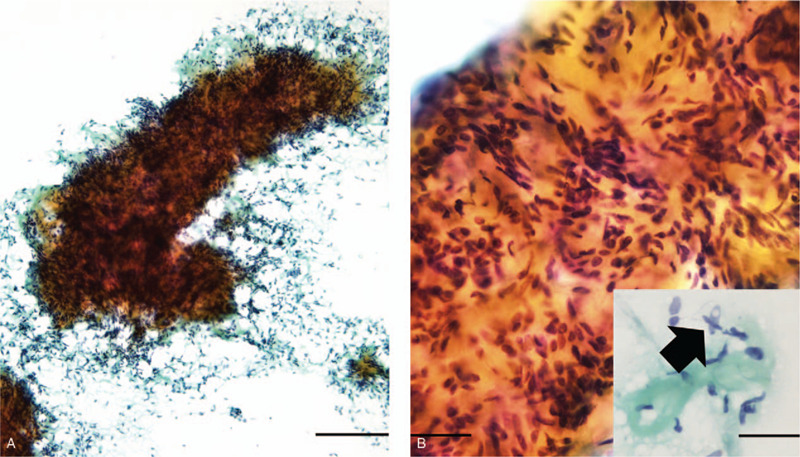
Cytological findings. (A) A large and nodular cell cluster was found within the background of myoepithelial cells. (B) Slightly atypical spindle-shaped or round cells with occasional intranuclear inclusion bodies were seen (inset arrowhead). Bar A: 100 μm, B: 20 μm.

A 64-year-old woman presented with a mass, which had been observed for 10 years at her local clinic, in the lower outer region of her right breast. Mammography showed focal asymmetric density, corresponding to category 3. Ultrasonography revealed multiple hypoechoic masses. Magnetic resonance imaging (MRI) showed a modular lesion with calcification, measuring 30 × 30 mm in size, in the lower outer region of the right breast (Fig. 1). Fine-needle aspiration cytology revealed slightly atypical spindle-shaped and/or round cells with intranuclear inclusion bodies (Fig. 2A and B), which resulted in a diagnosis of an indeterminate lesion that was suspected to be a spindle cell tumor. A biopsy examination revealed that the lesion was composed of spindle-shaped and/or round cells. Immunohistochemically, these cells were positive for AE1/AE3 and cytokeratin (CK) 5/6 and partially positive for p63 and calponin. AME was suspected, and lumpectomy was performed.

**Figure 3:**
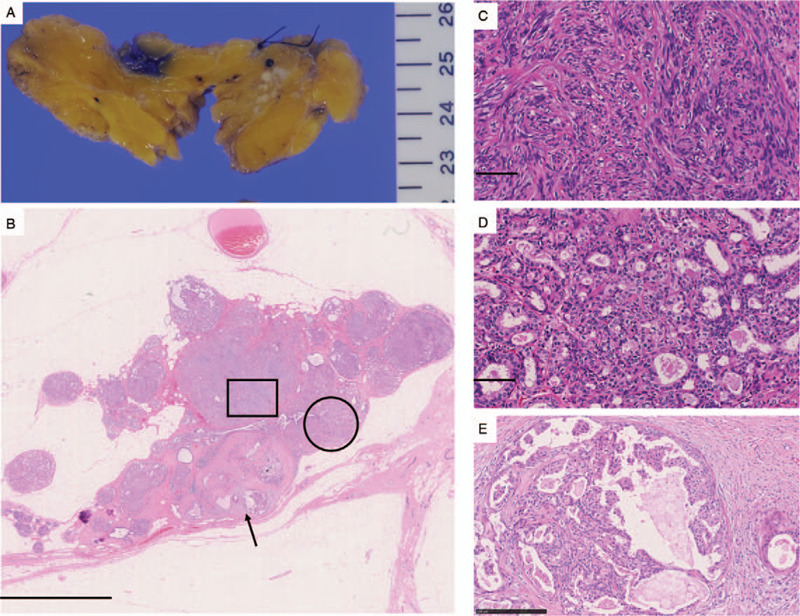
Lumpectomy findings. (A) A gross view of the tumor showed a well-defined, firm, whitish multinodular lesion. (B) A whole histological section of the tumor showed that it consisted of a spindle cell lesion (square), a tubular epithelial lesion (circle), and an intraductal component (arrowhead). (C) The spindle-shaped tumor cells only exhibited slight nuclear atypia. (D) The tumor cells in the tubular epithelial lesion also only showed slight nuclear atypia. (E) The intraductal lesion demonstrated a papillary or cribriform growth pattern. The tumor cells of the intraductal lesion displayed increased nuclear atypia. Bar B, 2.5 mm; C and D, 100 μm; E, 250 μm.

Macroscopically, the resected tumor was a well-defined, firm, whitish multi-nodular lesion (Fig. 3A). Microscopically, the tumor consisted of 3 components: a spindle cell lesion, a tubular lesion, and an atypical intraductal proliferative lesion. The spindle cell lesion, which accounted for most of the tumor, consisted of spindle-shaped myoepithelial cells with small to medium-sized nuclei and slight atypia (Fig. 3B and C). The tubular lesion comprised part of the tumor. In this region, ductal cells that exhibited only slight nuclear atypia grew in a tubular pattern and were surrounded by myoepithelial cells (Fig. 3B and D). In addition, an atypical intraductal proliferative lesion, which displayed papillary and cribriform patterns, was also seen in a small area of the tumor (Fig. 3B and E). The intraductal proliferative lesion displayed increased nuclear atypia. No mitoses or necrosis was observed in any of the 3 components.

**Figure 4:**
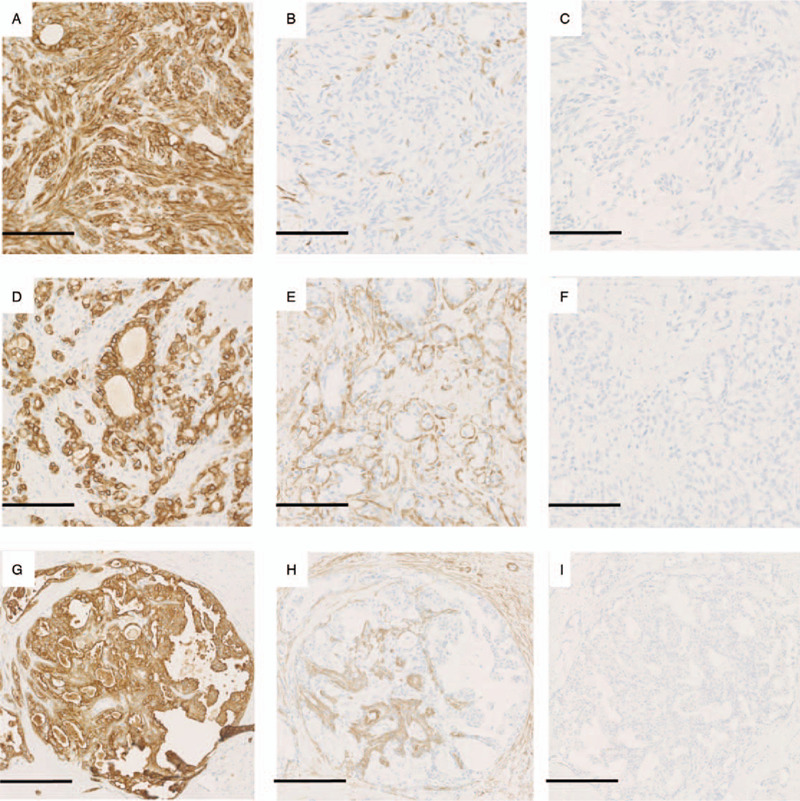
Immunohistochemical findings. The spindle cell component was positive for AE1/AE3 (A), focally positive for SMA (B), and negative for the ER (C). The tubular luminal lesion was positive for AE1/AE3 (D), negative for SMA (only the outer myoepithelial cells were positive for SMA) (E), and negative for the ER (F). The intraductal component was positive for AE1/AE3 (G), negative for SMA (only the outer myoepithelial cells of the intraductal component were positive for SMA) (H), and negative for ER (I). Bar A–F: 100 μm, G–I: 250 μm. ER = estrogen receptor; SMA = smooth muscle actin.

The immunohistochemical results are summarized in Table 1. The spindle cell lesion was positive for AE1/AE3 (Fig. 4A) and CK7 and focally positive for CK5/6, while myoepithelial markers, smooth muscle actin (SMA) (Fig. 4B), calponin, and p63, were only expressed at minimal levels. The tubular lesion and atypical intraductal proliferative lesion were positive for AE1/AE3 (Fig. 4D and G) and CK7, and focally positive for CK5/6. The outer myoepithelial component, which was positive for calponin (Fig. 4E and H) and SMA, was present in these lesions.

**Table 1:**
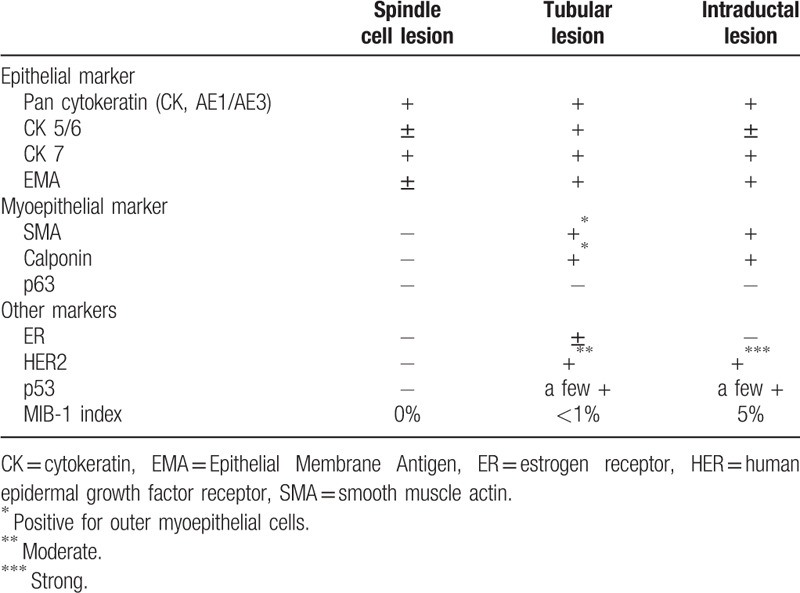
Immunohistochemical profiles of the present case.

**Figure 5:**
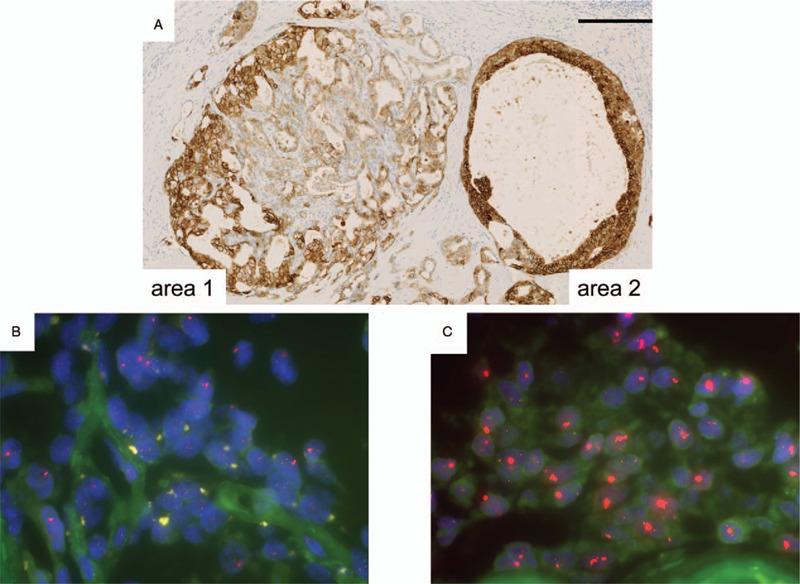
HER2 immunohistochemistry and HER2 FISH. (A) HER2 immunoreactivity varied from equivocal (area 1) to positive (area 2) in the tubular lesion and atypical intraductal proliferative lesion, respectively. HER2 FISH showed no amplification (HER2/CEP17: 1.48) in area 1 (B), but clear amplification (HER2/CEP17: 6.0) in area 2 (C). Bar A: 250 μm. CEP = chromosome enumeration probes, FISH = fluorescence in situ hybridization, HER = human epidermal growth factor receptor.

The spindle cell, tubular, and atypical intraductal proliferative lesions were negative for the estrogen receptor (ER) (Fig. 4C, F, and I) and progesterone receptor (PgR). None of the lesions exhibited p53 overexpression or a high MIB-1 index. Membranous HER2 immunoreactivity, which varied from moderate (Fig. 5A, area 1) to strong (Fig. 5A, area 2) in intensity, was observed in the tubular and atypical intraductal proliferative lesions. The findings of the atypical intraductal proliferative lesion were suggestive of DCIS, but they were not conclusive. Therefore, we performed dual-probe FISH analysis using locus-specific HER2 and centromere enumeration probes (CEP17). We examined 2 areas of the atypical intraductal proliferative lesion. One area, in which HER2 immunoreactivity of moderate intensity was seen, showed no amplification (HER2/CEP17 = 1.48) (Fig. 5B). The other area, in which strong HER2 immunoreactivity was observed (Fig. 5C), showed HER2 amplification (HER2/CEP17 = 6.0). Thus, we determined the latter to be a DCIS component. As the spindle cell lesion only exhibited minimal staining for myoepithelial markers, we considered that our case might correspond to AME, spindle-cell type.^[7]^ Thus, we eventually diagnosed the patient with AME (spindle-cell type) with DCIS.

The patient did not receive any further treatment and was free from tumor recurrence at 23 months after the operation.

## 3 Discussion

In this report, we described a case of AME of the breast, which mainly consisted of spindle-shaped cells with only slight atypia, admixed with tubular luminal cells and small atypical intraductal proliferative lesions. The latter lesions had features that were suggestive of DCIS, but lacked definitive features of malignancy, such as necrosis and invasion. As HER2 amplification was demonstrated by HER2 FISH, we made a final diagnosis of AME with DCIS.

The clinicopathological findings of 17 cases of AME with non-invasive carcinoma, including our case, are summarized in Table 2.^[3,4,6–16]^ The disease did not exhibit and predilection for either side of the body (left-sided cases: 5/16, right-sided cases: 8/16, unknown cases: 3/16). The mean tumor size was 34.7 mm (range: 3–150 mm), and the mean age at onset was 55.6 years (range: 39–86 years). Our case is largely consistent with the findings of previous reports with regard to tumor size and age at onset. The previously reported patients had relatively good prognoses, except in 2 cases. In 1 of these cases, the tumor was large (150 mm in diameter),^[14]^ whereas in the other case the tumor measured 40 mm in diameter.^[13]^

**Table 2:**
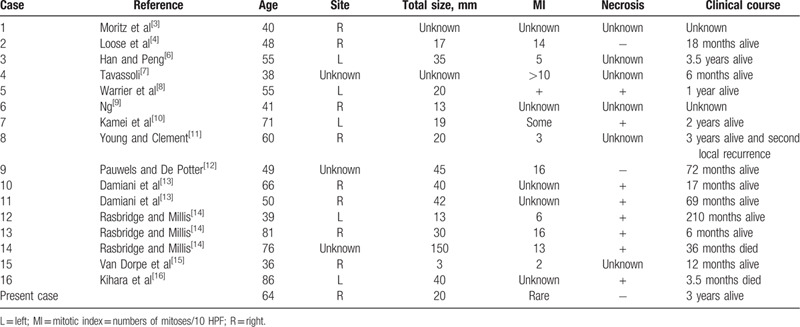
Pathological and clinical features of cases of AME of the breast with non-invasive carcinoma reported in the literature and the present case.

There were no descriptions of the relative sizes of the atypical/malignant components in AME. In our case, the DCIS component was localized in a small area (1 mm in size). Histologically, many of the reported cases exhibited mitotic figures (68.8%, 11/16) or necrosis (50%, 8/16). In these cases, the diagnosis of malignancy was made easily.

No previous reports have described the HER2 expression status of AME in detail. The other reported cases of AME and malignant AME were negative for HER2, except for 1 case.^[17]^ Therefore, our case might be unique with regard to its HER2 expression status, and this is the first case report of AME with DCIS, in which HER2 FISH was the key to establishing the diagnosis.

Genetically, AME has a heterogeneous ER status. ER-positive AME exhibited phosphatidylinositol-4,5-bisphosphate 3-kinase, catalytic subunit alpha (PIK3CA) or RAC-alpha serine/threonine-protein kinase (AKT1)-activating mutations, whereas ER-negative AME expressed recurrent GTPase HRas (HRAS) mutations, which co-occurred with PIK3CA or phosphatidylinositol 3-kinase regulatory subunit alpha (PIK3R1) mutations.^[18]^ Neither type demonstrated HER2 gene amplification.^[18]^ AMEs and their respective carcinomatous or metastatic components displayed HRAS Q61 hot-spot mutations, PIK3CA mutations, PIK3R1 mutations, and cyclin-dependent kinase inhibitor 2A (CDKN2A) homozygous deletions, whereas telomerase reverse transcriptase (TERT) promoter mutations might constitute early or late events in the development and/or progression of AME.^[18]^

In DCIS, the significance of HER2 amplification remains unclear and might depend on various parameters.^[19–22]^ Unlike in invasive carcinoma, the significance of the molecular phenotypes of DCIS remains unclear.^[9]^ According to one study, the frequency of recurrence at 5 years was low in luminal A-type (ER/PgR+HER2-) DCIS, whereas it was high in HER2-type (ER and PgR-/HER2+) DCIS.^[19]^ Our case corresponds to HER2-type DCIS. Therefore, the patient might need careful follow-up.

In conclusion, we have reported for the first time a rare case of AME with a DCIS component, which was confirmed by HER2 FISH analysis. As this is a report of a single case, molecular studies of a large case series are needed to clarify the significance of HER2 amplification in malignant AME.

## Author contributions

**Conceptualization:** Yusuke Amano and Toshiro Niki.

**Data curation:** Yusuke Amano, Mio Sakaguchi-Tamba, Yumiko Sasaki, Hisashi Oshiro, Noriyoshi Fukushima, and Takashi Fujita.

**Formal analysis:** Yusuke Amano, Mio Sakaguchi-Tamba, Hisashi Oshiro, Noriyoshi Fukushima, Shinobu Masuda, and Toshiro Niki.

**Investigation:** Yusuke Amano, Mio Sakaguchi-Tamba, Hisashi Oshiro, Noriyoshi Fukushima, Shinobu Masuda, and Toshiro Niki.

**Methodology:** Yusuke Amano and Toshiro Niki.

**Project administration:** Yusuke Amano.

**Resources:** Yusuke Amano, Mio Sakaguchi-Tamba, Yumiko Sasaki, Hisashi Oshiro, Noriyoshi Fukushima, and Takashi Fujita.

**Software:** Yusuke Amano.

**Supervision:** Toshiro Niki.

**Validation:** Yusuke Amano, Mio Sakaguchi-Tamba, Hisashi Oshiro, Noriyoshi Fukushima, Shinobu Masuda, and Toshiro Niki.

**Visualization:** Yusuke Amano.

**Writing – original draft:** Yusuke Amano.

**Writing – review & editing:** Yusuke Amano, Hisashi Oshiro, and Toshiro Niki.
